# A213 DUPILUMAB EFFICACY IN EOSINOPHILIC ESOPHAGITIS PERSISTS FOR HISTOLOGIC, SYMPTOMATIC, AND ENDOSCOPIC OUTCOMES REGARDLESS OF CONCOMITANT HIGH-DOSE PROTON PUMP INHIBITOR USE

**DOI:** 10.1093/jcag/gwad061.213

**Published:** 2024-02-14

**Authors:** M Rothenberg, E Dellon, A J Bredenoord, E Martire, X Sun, E Laws, E Mortensen, J Maloney, L Glotfelty, A Shabbir

**Affiliations:** Cincinnati Children’s Hospital Medical Center and University of Cincinnati College of Medicine, Cincinnati, OH; University of North Carolina School of Medicine, Chapel Hill, NC; Academic Medical Centers, Amsterdam, Netherlands; Sanofi, Mississauga, ON, Canada; Regeneron Pharmaceuticals Inc., Tarrytown, NY; Sanofi, Bridgewater, NJ; Regeneron Pharmaceuticals Inc., Tarrytown, NY; Regeneron Pharmaceuticals Inc., Tarrytown, NY; Sanofi, Bridgewater, NJ; Regeneron Pharmaceuticals Inc., Tarrytown, NY

## Abstract

**Background:**

Proton-pump inhibitor (PPI) therapy is the most commonly used first-line therapy for eosinophilic esophagitis (EoE) but data informing long-term outcomes are limited.

**Aims:**

This pre-specified analysis of data from the phase 3 LIBERTY EoE TREET (NCT03633617) study assessed the efficacy of weekly dupilumab vs placebo in patients (pts) with and without concomitant PPI use.

**Methods:**

Pts with peak intraepithelial eosinophil (eos) count ≥15 eos/high-power field (hpf) after ≥8 weeks’ high-dose PPI were randomized to dupilumab or placebo. Pts on high-dose PPIs at screening remained on a high-dose regimen during the treatment period; switching of PPI types was permitted but new initiation of PPIs was prohibited. Endpoints at Week 24 were: proportion of pts achieving ≤6 eos/hpf, absolute change from baseline in Dysphagia Symptom Questionnaire (DSQ) score, % change in peak eos count, absolute change in Endoscopic Reference Score (EREFS) and Histologic Scoring System (HSS) grade/stage scores.

**Results:**

In Parts A and B, respectively, 61.9% and 71.3% of pts in the dupilumab group and 64.1% and 72.2% in the placebo group were using PPIs at randomization. For pts treated with dupilumab vs placebo in Parts A and B, respectively, ≤6 eos/hpf was achieved by 69.2% (95% confidence interval [CI] 48.2–85.7) vs 8.0% (95% CI 1.0–26.0) and 59.6% (95% CI 45.8–72.4) vs 7.0% (95% CI 2.0–17.0) of pts using PPIs, and by 43.8% (95% CI 19.8–70.1) vs 0% (95% CI 0–23.2) and 56.5% (95% CI 34.5–76.8) vs 4.5% (95% CI 0.1–22.8) of pts not using PPIs. Least squares mean change from baseline in DSQ score for dupilumab vs placebo was –18.5 vs –12.9 and –21.8 vs –14.0 for pts using PPIs in Parts A and B, respectively, and –27.8 vs –3.8 and –28.4 vs –13.3 for those not using PPIs (**Figure 1**). Dupilumab improved outcomes vs placebo for secondary endpoints, with comparable results in pts with and without concomitant PPI use. Absolute change from baseline in EREFS score is shown in **Figure 1**. Dupilumab was generally well tolerated.

**Conclusions:**

Dupilumab improved histologic, symptomatic, and endoscopic aspects of EoE in adults and adolescents, up to 24 weeks, regardless of concomitant high-dose PPI use.

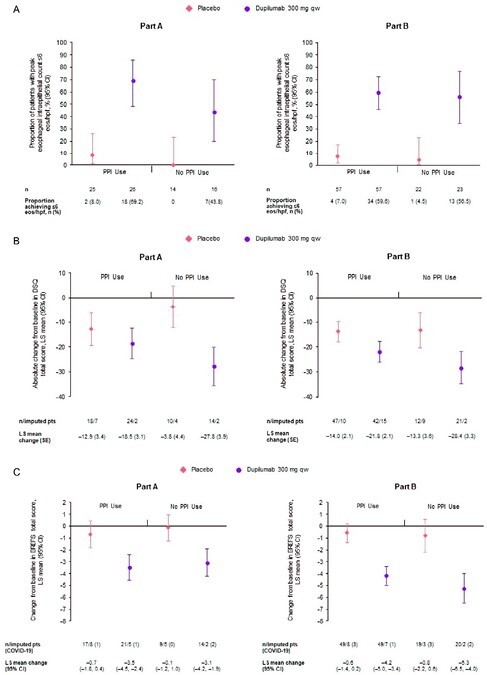

**Figure 1.** Effect of dupilumab 300 mg qw versus placebo on primary endpoints (A) proportion of patients with peak esophageal intraepithelial eosinophil count of ≤6 eos/hpf (B) absolute change from baseline in DSQ total score at Week 24 (C) absolute change from baseline in EREFS total score at Week 24, by concomitant PPI use

CI, confidence interval; DSQ, Dysphagia Symptom Questionnaire; EREFS, endoscopic reference score; eos, eosinophils; hpf, high-power field; LS, least squares; PPI, proton pump inhibitor; pt, patient; qw, once weekly; SE, standard error.

**Funding Agencies:**

Research sponsored by Sanofi and Regeneron Pharmaceuticals Inc.

